# Use of web mining in studying innovation

**DOI:** 10.1007/s11192-014-1434-0

**Published:** 2014-09-12

**Authors:** Abdullah Gök, Alec Waterworth, Philip Shapira

**Affiliations:** 1 Manchester Institute of Innovation Research, Manchester Business School, University of Manchester, Manchester, UK; 2 Georgia Institute of Technology, Atlanta, GA USA

**Keywords:** Web mining, Web scraping, Innovation, R&D

## Abstract

As enterprises expand and post increasing information about their business activities on their websites, website data promises to be a valuable source for investigating innovation. This article examines the practicalities and effectiveness of web mining as a research method for innovation studies. We use web mining to explore the R&D activities of 296 UK-based green goods small and mid-size enterprises. We find that website data offers additional insights when compared with other traditional unobtrusive research methods, such as patent and publication analysis. We examine the strengths and limitations of enterprise innovation web mining in terms of a wide range of data quality dimensions, including accuracy, completeness, currency, quantity, flexibility and accessibility. We observe that far more companies in our sample report undertaking R&D activities on their web sites than would be suggested by looking only at conventional data sources. While traditional methods offer information about the early phases of R&D and invention through publications and patents, web mining offers insights that are more downstream in the innovation process. Handling website data is not as easy as alternative data sources, and care needs to be taken in executing search strategies. Website information is also self-reported and companies may vary in their motivations for posting (or not posting) information about their activities on websites. Nonetheless, we find that web mining is a significant and useful complement to current methods, as well as offering novel insights not easily obtained from other unobtrusive sources.

## Introduction

Enterprises use their publicly-viewable websites for a variety of reasons, including promoting their products and services, directly selling those products and services, presenting information about their development, capabilities and credentials, documenting their achievements, and expanding their customer base, especially in export markets (Fisher et al. 2007). Enterprise websites often also contain valuable information about the company’s location(s) and facilities, specifications of products and services offered, the orientation and attitude of the firm, key personnel, and strategies and relationships with other firms and organizations.

The ever-growing amount of information that is available through enterprise websites offers significant opportunities for researchers. With the understanding that websites are self-reports, website information has advantages in that it is readily and publicly available, is cost-effective to obtain, and can be extensive in terms of coverage and the amount of data contained. In an era where corporate response rates to voluntary academic research questionnaire surveys are frequently rather low, those same corporations maintain a relatively high website presence. For example, just under three-quarters of UK companies with at least one employee indicate that they maintain a website—a figure that rises to 85 % for companies (all sectors) with 10 or more employees and 91 % for manufacturing companies with 10 or more employees.^1^

We recognize that the information that is available on enterprise websites is not standardized, varies according to the company and how it wishes to present itself, and is typically in an unstructured format. Yet, notwithstanding these and other caveats, we suggest that the data that can be found on enterprise websites is an additional and important source of information and intelligence, particularly in addressing questions related to innovation where other data sources are less effective in gathering sufficient and relevant information. Technical advances in handling and analyzing unstructured data as well as current interest in the use of “big data” in discovering patterns and trends make it timely to investigate the appropriateness of using enterprise websites as a data source. However, while there are significant benefits to using website data through methods such as web scraping or web mining in innovation research, the literature on the use and validity of these approaches is relatively underdeveloped. This article aims to address this issue by analyzing the usefulness of website data in comparison with other data sources. We explore a web-derived dataset to discuss methodological issues related to the processes of conceptualizing, retrieving, structuring, cleaning, manipulating and interpreting website data in understanding company innovation strategies, focusing on enterprise research and development (R&D) activity.

In the next section, we review the available literature on the use of website data in innovation studies and wider social science applications. This is followed by discussion of our sample dataset and the methodologies used to obtain and analyze the data. In the ensuing section, there is an empirical demonstration of how the web mining process is operationalized in identifying R&D activities, as well as how this data correlates with other data sources. We then present a conceptual discussion of the relative qualities of website data for evaluating the enterprise R&D activities in comparison with other data sources. A discussion of the results and conclusions, as well as limitations, is contained in the final section.

## Literature review

Researchers often select methods such as web mining due to their “unobtrusiveness”. Webb et al. (1966) first coined the term “unobtrusive measures” in reference to methods of data collection that do not require direct contact with research subjects. Conversely, obtrusive methods can be regarded as those that require direct contact with the population studied.

These methods are each suitable to different circumstances, depending on what is being studied. For example, the opinions and beliefs of individuals are often best explored through interviews or questionnaires—obtrusive methods. However, if the research concerns real actions and behaviors, these may best be observed from a distance—using unobtrusive methods. Unobtrusive methods are a way of collecting data about a subject without their direct knowledge or participation (Cargan 2007). Unobtrusive methods can be less expensive in that they do not involve the costs of training and placing researchers in the field and following up directly with respondents. Additionally, as Lee (2000) discusses, one major advantage of using “non-reactive” approaches (Webb et al. 1981) is that they avoid problems caused by the researcher’s presence. In the case of obtrusive methods, the respondents are aware of the researcher and may alter their response to these research methods in light of this. Unobtrusive methods are also not limited to those who are accessible and cooperative (Webb et al. 1966). Lee (2000) also outlines the opportunity that internet data presents in unobtrusive research.

In the field of innovation studies, there has long been the use of a combination of obtrusive methods (such as innovation surveys of firms or business case studies) and unobtrusive methods (such as analyzing databases of patents and publications). More recently, innovation researchers have demonstrated increasing creativity in developing more diverse unobtrusive methods, many of which use website or social media data. Robson (2002) distinguishes between the more-traditional unobtrusive approaches already discussed and another—content analysis. The author describes content analysis as that conducted on a written document, such as books, letters and newspapers. However, we can see how this can be extended to analyzing the textual content of a website, through the process of web mining. Robson states that such an approach is different from other unobtrusive methods as the observation itself is indirect (i.e. there is no need to observe the participants – in our case, a group of companies—directly).

There are three general categories of web mining (Miner et al. 2012). *Web content mining* involves the analysis of unstructured text data within webpages to extract structured information. *Web structure mining* focuses on analyses of the hyper-linked structure of a set of webpages, typically using methods of network analysis. *Web usage mining* is the data mining process involving the usage data of webpages. All three types of web mining have been used in innovation studies.

An example of web structure mining in innovation studies is offered by Katz and Cothey (2006) who investigate relationships between the internet and innovation systems by utilizing website-based indicators from webpage counts and links. Another instance of web structure mining is from van de Lei and Cunningham (2006), who employ website data in a future-oriented technology analysis, where it is used to identify existing networks that are concerned with technological change. In this research, a web crawling process is used to identify linkages between nanotechnology web portals, creating a network of activity between parties across many sectors. Ladwig et al. (2010) use web structure mining to study the landscape of online resources in emerging technologies by identifying the top search terms and resulting top-ranked webpages from Google. Similarly, Ackland et al. (2010) use web crawling to capture hyperlinks: examining the relationships between, and prominence of, actors engaged in nanotechnology. The use of metrics based on web presence in measuring scientific performance (“webometrics”) has widely been discussed in science policy literature (see Thelwall (2012) for an overview). Webometrics approaches use both web structure mining and web usage mining.

More recently, innovation scholars have been applying web content analysis in their research. Veltri (2013) carried out semantic analysis on 24,000 tweets from Twitter to understand the public perception of nanotechnology. Libaers et al. (2010) examine keyword occurrence in company websites from a cross-industry sample of small and medium-size enterprises to identify commercialization-focused business models among highly-innovative firms. Hyun Kim (2012) conducted both web-content and web-structure analysis of nanotechnology websites across the “Triple Helix” (Etzkowitz and Leydesdorff 2000) of university, government and enterprise relationships. The former allowed the author to discern different lexicons from three sectors, while the latter offered an understanding of which organizations played key roles in the development of an emerging technology.

Two recent studies are notable for examining the commercialization of emerging technologies by small and medium-sized firms through web content analysis. Youtie et al. (2012) examine current and archived website data of nanotechnology small and medium-sized enterprises, with a particular focus on the transition of such technologies from discovery to commercialization. The authors note the problems of coverage, timeliness, and response rate in commonly used sources of information such as patent databases and surveys in understanding enterprise innovation in rapidly transforming domains. A new approach—one which uses current and archival website data—is proposed. This method involved identifying and mining content information found on the websites of a pilot sample of 30 small and medium-sized enterprises from the United States, then analyzing the unstructured data in order to draw findings. The authors note that smaller firms tend to have smaller websites, therefore making the web mining process and subsequent analysis more manageable in such cases. From their analysis of the website data, the authors were able to identify the occurrence of various innovation stages and production transitions in the development of their sample of enterprises. The paper also discusses the role of government research grants and venture capital investment in bringing a technology to market.

The second study by Arora et al. (2013) undertakes a similar web content analysis method to examine the activities of small and medium size enterprises in the US, UK and China commercializing emerging graphene technologies. The authors again discuss the limitations of conventional methods, including issues of response rate and bias in surveys, and coverage and time lag in bibliometric and patent data. The study employs a web crawling technique of searching for keywords across all webpages of the sample firms’ websites. This allowed the authors to not only draw conclusions on the degree of innovation employed by the sample firms but also the extent to which these activities were globalized and in partnerships, using such analysis to characterize three different types of emerging technology SME.

Web mining has also been used in other areas of social science. AleEbrahim and Fathian (2013) develop a method to summarize customer online reviews from websites. Al-Hassan et al. (2013) investigate whether the North American Industry Classification System code (NAICS) effectively shows the true industrial sectors of Fortune 500 firms by analyzing their websites. Battistini et al. (2013) present a technique to map geo-tagged geo-hazards, such as landslides, earthquakes and floods, by analyzing online news. Hoekstra et al. (2012) investigate the feasibility and desirability of the automated collection of official statistics, such as consumer price index, from websites. There is a stream of publications concerning the mining of political opinions from websites, forums and social media (Sobkowicz et al. 2012; Sobkowicz and Sobkowicz 2012). There are also attempts to use web mining in health research: for instance content mining of website discussion forums to detect concern levels for HIV/AIDS (Sung et al. 2013) and mining social media to discover drug adverse effects (Yang et al. 2012).

## Research questions

In this study, we develop and operationalize a web content mining process to assess the extent and character of R&D activities in a sample of small and medium sized enterprises. We discuss the specific process of extracting keywords for this purpose and explore how the final results are sensitive to the procedure used. We also compare and contrast the effectiveness of website data to other data sources in studying the innovation process. In summary, the key research questions of the study are:

- How can web content mining be operationalized to study business R&D activities?
- How sensitive are the results to the web content mining procedure followed?
- How do website-based R&D activity indicators compare with other conventional R&D indicators?
- What are the relative advantages and disadvantages of website data over other data sources for understanding enterprise-level R&D activities?

## Data and methodology

The data we use in this study is derived from the project on Sustaining Growth for Innovative New Enterprises, which is examining the determinants of growth in green goods small and medium-sized enterprises (Shapira and Harding 2012). Green goods firms are enterprises who are involved in the production of manufactured goods whose outputs benefit the environment or conserve natural resources. These firms, who produce goods for use in renewable energy, environmental control, and other low carbon applications, typically develop and employ a range of innovative technologies. The project is focusing on small and medium-size enterprises, started in the last decade or so, to probe how such firms grow, what strategies and relationships are employed, and what is the role of external policy and regional resources in supporting growth. To address the limitations of conventional approaches for identification of firms (such as government statistical industrial classifications, which generally lag in developing classifications for emerging new technology sectors), the project developed a comprehensive set of green goods search terms, applicable across sectors, which identified qualifying firms when extracted through textual searches of the business descriptions (for full details of this method, see Shapira et al. 2014). Following a text mining search of the FAME (2014) enterprise database (which incorporates data from UK official company registrations), and subsequent manual validation, a sample of 296 UK-based green goods firms was identified. For the timeframe of 2004–2012, multiple items of data were combined together for each identified green goods firm, drawing from the following data sources:

1. The FAME time series enterprise database for financial information, including R&D expenditure (FAME, 2014).
2. The Technology Strategy Board (TSB) funded R&D projects database, which includes the extent to which identified firms receive support from the UK government for their R&D activities.
3. Publications and patents from the Scopus and Derwent databases.
4. Firm websites (current and archived websites).

A set of keywords was developed for the variables that were to be measured by the web content mining process, subsequently arranged into variable sets and subsets. These included manufacturing strategy (products, manufacturing intensity, customization, greenness); linkages (universities, partnerships, membership organizations, regional/extra-regional links); investment strategy (venture capital, investment); policy influence (regulation); and R&D activity (R&D, research, development, product development, technology development, and related terms). About 25 of the firms are also the subject of case studies to compare insights gained from web and other unobtrusive data sources with those obtained from interviews.

In this paper, we concentrate particularly on variables related to R&D, obtained from the different data sources mentioned above and compare them to indicators derived from the firm websites. From the FAME database, we use company reported R&D expenditures. The TSB database provides us with R&D financial awards from UK governmental sources. Publications and patents for the companies are drawn from the Scopus and Derwent databases. Additional information about company R&D activities is derived from firm websites.

## Operationalization

This section discusses the operationalization of the web content mining process used to understand the R&D activities of the firms in our dataset. We discuss the mechanics of the data collection retrieval, preparation and cleaning. The procedure to transform the unstructured website data into structured data is also discussed, as well as the sensitivity analysis conducted on a number of keyword sets.

### Operationalization of web content mining

After identifying target firms, the first step in the process is web crawling, an automated process used to capture web pages for subsequent analysis. The web crawling process was conducted in IBM Content Analytics (ICA). We started with a seed list of website domain addresses available in the FAME database. We manually checked and cleaned these website addresses against misspelling and address changes, dropping dead links and adding a small number of new addresses. The resulting seed list of website addresses was fed into the ICA crawler. Once the crawler started, it progressively advanced from the initial seed website address to all other subdomains and webpages on that website, capturing all accessible text. This included text residing within HTML (HyperText Markup Language) as well as other text-based files such as Portable Document Format (PDF) and word processing (DOC and DOCX) files. At the time of the analysis, 2012 was the current or live year. Web pages for the years 2004–2011 were accessed through the Internet Archive Wayback Machine (http://archive.org/web/)—an online archive of past instances of websites. One limitation of using the Wayback Machine to access archived legacy websites is that the Wayback Machine tends to have better coverage of larger websites (which are inclined to be associated with larger firms). Additionally, Wayback Machine coverage is better in some years than in other years. At the end of this stage, rules were set to exclude certain web addresses from the collection. This process encompassed excluding irrelevant sub-domains of a website, which was useful in isolating an English-language version of a website (e.g. allowing www.companyname.com but excluding fr.companyname.com) and in restricting irrelevant sections of large websites (e.g. allowing www.companyname.com but excluding forum.companyname.com). For 2012, we identified 237 firms with websites, out of our initial sample of 296 firms – equivalent to a response rate of 80.1 %. The number of firms with web sites diminishes for earlier years. The number of firms with websites, number of webpages and total number of phrases for each year is presented in Table 1.

**Table 1:** Firms and Data Included in the Web Content Analysis

Year	Firms with websites	Webpages (thousands)	Phrases (millions)
2004	125	14.9	1.9
2005	133	11.7	2.0
2006	131	15.8	2.0
2007	173	10.6	1.3
2008	173	12.8	1.2
2009	161	10.8	1.3
2010	163	13.0	1.6
2011	199	15.8	2.6
2012	237	51.7	10.3

*Source*: Website analysis of sample of 296 UK-based green goods small and medium-sized enterprises (see text for details)

Once crawling was completed on all webpages within a collection (i.e. year), the data collected in that collection was indexed and structured into a number of fields. The data was exported from ICA as a series of XML (Extensible Market Language) files that were both human and machine readable. The XML files were then imported into VantagePoint—a software tool for text mining and analysis. In the course of carrying out this process, a single year was crawled a number of times, as and when problems emerged with particular web sites. In such cases, these datasets were merged and duplicates were removed, resulting in a single and complete dataset for each year. From here, the data was cleaned—a laborious but important part of the process. A number of errors were flagged and corrected, for example: webpages that were not assigned to a firm; webpages not in the English language; duplicated webpages; and irrelevant or non-pertinent webpages, such as customer forums or non-germane sub-domains.

Following this cleaning process, our variables were extracted from the dataset, having first being imported from a group of text files. A further, final cleaning process was required at this stage, for cases where false positives were captured by the extracted variables. For example, the word *machine* (included as part of a keyword set measuring manufacturing intensity) delivered false positives from the past year collections that were drawn from the Wayback Machine (as Wayback Machine included a header for all webpages). We developed a procedure to exclude such references. Upon completion of this final cleaning process, the dataset was exported as a structured data set. The output was a table of firms against variables (either individual keywords or variable sets). Both the number of occurrences, with counts of multiple instances on the same webpage, and the number of webpages were tallied. The total number of words and webpages for each firm within the dataset was also captured to allow for a normalization process. The data set was then structured and ready to be imported to STATA—a widely used statistical analysis software package.

**Fig. 1:**
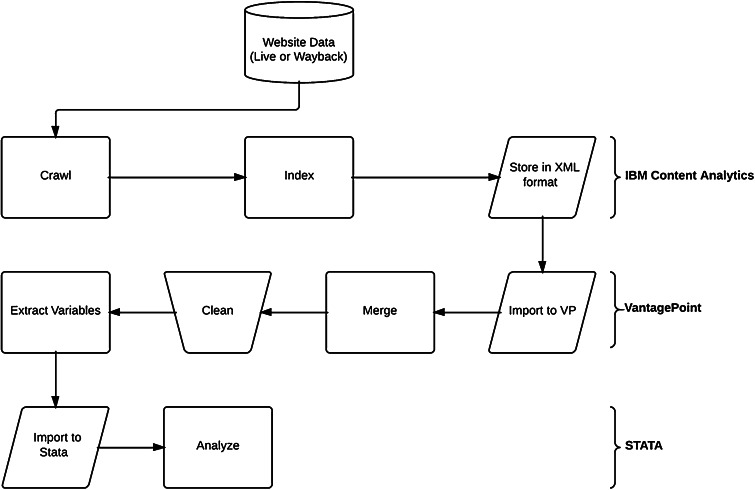
Web content analysis process

In STATA, we experimented with a number of conversion and normalization operations. We observed that there was a high degree of variety in terms of the size of websites. Some firms had websites with only a few webpages as a placeholder, whilst others built extensive websites with thousands of webpages. Therefore, normalization of the variables captured was required. After trying both the number of webpages and the number of noun phrases for normalization of variables, we decided to use the latter as the former was not suitable to the variation of the number of webpages across the sample. We then conducted a stock-flow conversion process. The number of keyword occurrences is a flow variable and may not be suitable to be used in conjunction with flow variables in time series data. Therefore, we created flow variables by using the year-on-year percentage changes (Fig. 1).

### Defining the keywords for R&D activity

To capture R&D activity, we conducted a simple rule-based variable analysis, structured into six different variable sets. This is summarized in Table 2 in Boolean format. These definitions of R&D activity emerged through an iterative sensitivity analysis. Starting with *rndweb1* variable, which simply identifies all webpages that include “research*”, we examined the results by manual inspection of the text on a sample of individual webpages and determined that it was far too limited. As a result, we iteratively added a further two keywords to subsequent variables. The addition of “development*” (to variable *rndweb2*) and “R&D” (to variable *rndweb3*) made a significant difference to our counts, particularly in the case of the former, although this was far from complete.

**Table 2:** R&D activity variables and keywords

R&D variable	Keywords	Difference from previous keyword set
rndweb1	Research*	
rndweb2	Research* AND development*	rndweb1 + (development*)
rndweb3	Research* AND development* AND R&D	rndweb2 + (R&D)
rndweb4	Research* AND development* AND R&D AND lab*, scientist*	rndweb3 + (lab, laboratory, scientist)
rndweb5	Research* AND (development NEARBY research) AND, R&D AND lab* AND scientist*	rndweb4 − (development [NOT NEARBY] research)
rndweb6	(Research and development) AND R&D AND lab* AND scientist* AND research AND researcher AND scientist* AND (product development*) AND (technology development*) AND (development phase) AND (technical development*) AND (development program*) AND (development process*) AND (development project*) AND (development cent*) AND (development facilit*) AND (technological development*) AND (development efforts) AND (development cycle) AND (development research) AND (research & development) AND (development activity)	rndweb5 − (development [NEARBY] research) + (a set of development variants)

We further examined a sample of webpages that had been captured by these additional keywords, which offered an insight into the words and phrases that appeared on the same webpage as these variables. As a result, a number of additional keywords were added to a fourth variable (*rndweb4*): “lab*, scientist*”. However, it was apparent that this variable was also capturing many false positives, particularly in relation to the keyword *“*development”. For instance, in our sample of green goods companies, there were a sizeable number of occurrences of *“*property development” that had no relationship to research and innovation activities. A further iteration (variable rndweb5) was undertaken, whereby rather than taking all instances of “development*”, we instead took only those where “development” appeared in the same sentence as “research” (a nearby phrase analysis). This again had a significant impact upon our counts for the keyword set. An examination of the rndweb5 variable, where “development” is captured nearby “research”, revealed that whilst this eliminates the capture of false positives of irrelevant “development” variants (e.g. “property development”), a number of relevant phrases were also left out in the process. Therefore, as a final step we developed the *rndweb6* variable, in which we explicitly defined a number of relevant instances that included the keyword “development”. This variable resulted in an optimum balance between capturing the essential concepts and keeping false positives to a minimum.

**Fig. 2:**
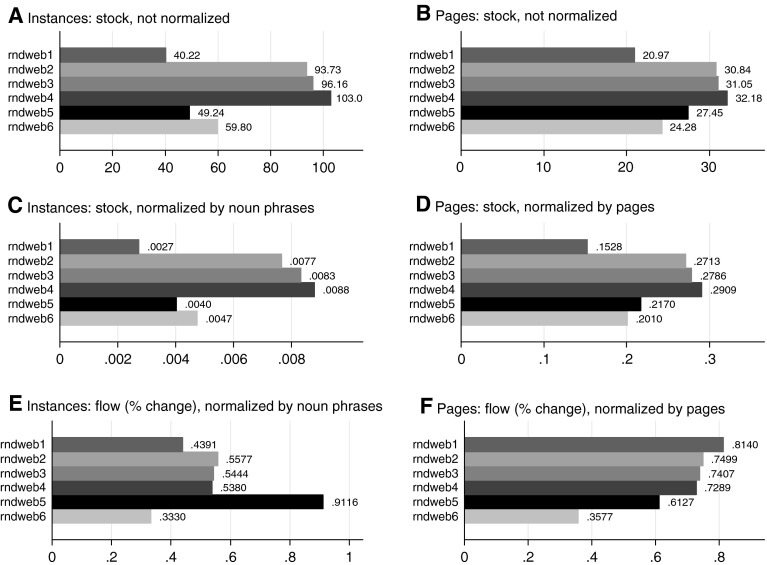
R&D website variables: comparison of mean values by different transformations and normalizations. *Source*: Analysis of website variables for sample of UK green goods small and medium enterprises. Mean values reported. Covers 2004–2012, see Table 1 for *N* each year. See Table 2 for keyword definitions of R&D variable labels

The six different R&D activity keyword sets performed differently in terms of results (Fig. 2). The average values for each keyword set across all firms and all years vary with a change in keywords. Furthermore, combinations of transformations (i.e. stock or flow treatment) and normalizations (i.e. noun phrases or webpage normalizations) changed the results significantly. An important conclusion from this process is that care needs to be taken in using simple keywords when mining web text, as false positives will likely be introduced. A more robust process is to ensure that keywords are used in the intended context, and to implement this with key phrases, context searches, and exclusion terms.

## Findings

This section presents findings and results from our analysis. This includes the relationship between the website-based R&D activity and other R&D indicators. We also discuss the relative qualities of different data sources in studying R&D.

### Results for R&D performance

We found that far more companies in our sample report on their websites that they undertake R&D activities than would be suggested by looking only at conventional data sources. For our sample of firms (*N* = 296), about 15 % patented at any time between 2004 and 2012, around 5 % published, around 17 % received an R&D grant and around 20 % reported R&D expenditure (according to enterprise reports captured by FAME). In contrast, the rndweb6 website-based variable finds that nearly 70 % of firms in the sample report some kind of research or development activity (including scientific research, technology development, and product development). For the full set of 2,664 firm observations over 9 years, patents cover 5.63 %, publications 1.24 %, grants 3.24 % and R&D expenditure data 7.02 %. However, the website based R&D activity variable (rndweb6) encompasses 34.12 % of all observations (Table 3).

**Table 3:** Coverage of website-based and other R&D variables

Variable	Explanation	Number of firms^c^	Coverage of firms (%)	Number of observations^d^	Coverage of observations (%)
Publications	Number of publications	43	14.5	150	5.6
Patents	Number of patents	15	5.1	33	1.2
R&D expenditure	Research and development spending^a^	51	17.2	91	3.4
Grants	TSB^b^ grant awards	66	22.3	187	7.0
rndweb6	Website-based variable, number of instances of keywords normalized by the number of noun phrases in websites	204	68.9	909	34.1

^a^R&D amount as reported in FAME (2014)

^b^TSB = UK Technology Strategy Board

^c^Firms reporting a value for this variable at any year between 2004 and 2012

^d^Non-missing observations over 9 years

While all five variables for R&D show the same phenomenon, the correlation between them is not highly significant. We conducted a simple pairwise cross correlation test at 95 % confidence level between these variables. The results show that there is a degree of correlation between R&D expenditure and grants and patents, although these are not correlated with publications. All four non-website-based variables are not correlated with the website-based variables at the 95 % significance level. However, website-based variables highly correlate with each other, due to the highly intersecting keywords sets used for these variables (Table 4). Overall, these results suggests that the web-site variables are providing additional information, and that many firms who report R&D-like activities on their websites do so without also seeking patents, publications, or R&D grants. The additional R&D website information presented by companies does not represent trade secrets, since by definition this is information that is not readily made public. Rather, it can be viewed as R&D that is company-sponsored, which the company cannot or is unable to patent, and which is likely to involve product and technological development rather than scientific research oriented towards publication.

**Table 4:** Pairwise 95 % significant correlations between website-based and other R&D variables

No	Variable	Explanation	1	2	3	4	5	6	7	8	9	10
1	Publications	Total number of publications	1									
2	Patents	Total number of patents		1								
3	R&D expenditure	Amount of R&D expenditure as reported in the FAME database, in GBP		0.5431	1							
4	Number of grants	Total number of grants from the TSB	0.9764	0.5367		1						
5	rndweb1	Website based variables, number of instances of keywords normalized by the number of noun phrases in websites					1					
6	rndweb2						0.8437	1				
7	rndweb3						0.8388	0.9974	1			
8	rndweb4						0.8265	0.9924	0.9957	1		
9	rndweb5						0.8317	0.8038	0.8230	0.8205	1	
10	rndweb6						0.8635	0.9108	0.9016	0.905	0.9551	1

*Source*: Analysis of conventional and website-based R&D variables for sample of 296 UK-based green goods small and medium-sized enterprises (see text for details)

### Comparing website data with other data sources

Website data has different characteristics compared to other data sources. The fundamental difference stems from the fact that website data is unstructured (i.e. there is no data schema). In contrast, financial databases most often provide structured data, in which variables are uniformly defined across all observations. Publications and patents are semi-structured in the sense that the data includes some structured variables, as well as some other variables consisting of text entries without a schema. The challenge of web content mining often lies in the process of structuring the unstructured data by creating a schema and processing the data in order to fit this schema.

We use and adapt the data quality framework from Batini and Scannapieco (2006) to compare the relative advantages and disadvantages of website data over other data sources, using the particular example of R&D activity. There are twelve data quality dimensions in Batini and Scannapieco’s framework. The relevance of these data quality dimensions to four different sources in our dataset are discussed below and summarized in Table 5.

**Table 5:** Comparison of different data sources

		R&D expenditure	R&D grants	Patents and publications	Analysis of R&D activity in websites
Source		Financial database (FAME)	Government database (TSB)	Web of science	Current and historic websites
Indicator type		Input	Input	Output	Process
Data structure		Structured	Structured	Semi-structured	Unstructured
Data quality dimensions^a,b^					
Completeness	Sufficient breadth and depth and scope	★☆☆	★☆☆	★☆☆	★★☆
Accuracy	Correct representation of the phenomenon	★☆☆	★☆☆	★☆☆	★★☆
Currency	How promptly data is updated	★☆☆	★★☆	★★☆	★★★
Volatility	Data change frequency	★☆☆	★★☆	★★☆	★★★
Consistency	Agreement among components	★★☆	★★★	★★☆	★☆☆
Interpretability	Easiness of interpreting meaning	★★★	★★★	★★☆	★☆☆
Accessibility	Easiness of access and analysis	★☆☆	★☆☆	★★☆	★★★
Handling	Easiness of analysis	★★★	★★★	★★☆	★☆☆
Amount	Quantity of data	★☆☆	★☆☆	★★☆	★★★
Flexibility	Adaptable to different purposes	★☆☆	★☆☆	★★☆	★★★

^a^Adapted and extended from Batini and Scannapieco (2006)

^b^Stars qualitatively denote the relative performance of data sources for data quality dimensions (three stars indicate relatively superior performance, while relatively inferior performance is denoted by one star). Comparison is made for the UK

#### Completeness (Coverage)

Website data promises advantages in terms of coverage of the population, as the majority of firms have websites. Financial, government support and publication and patent databases typically have lesser coverage. This is particularly important when research involves smaller size firms, which are not obliged to provide official financial data. Similarly, these smaller firms tend to publish very rarely and only a small percentage of them are likely to be supported by government grants. As with other data sources, website data can also cover multiple years, for example by using the Wayback Machine (with the limitation that use of the web first began to grow in the mid-1990s, with companies beginning to use web sites from the late-1990s and early 2000s onwards). In our sample, website data covers around five times more observations than financial data, 10 times more than administrative grants data and around seven times more than patent data.

#### Accuracy

Website data are self-reports, although this is also the case in one way or another for most other data sources. Keeping this in mind, website data has the potential to give a fuller picture in representing the broad extent of R&D activity. R&D expenditure data from the FAME database and the R&D support data from the TSB database are inputs for R&D activity. While this is often used as the main indicator of R&D activity, R&D inputs might not show the full extent of R&D activity, as some activities might be unfunded or firms may not keep a full account of their R&D expenditure (Kleinknecht et al. 2002). Similarly, patents and publications are output indicators of R&D activity and might miss the full extent of R&D activity. Only a small proportion of R&D activity might result in patent and publication outputs and even when they do so, firms may choose not to publish or patent for strategic reasons. (For a full discussion of advantages and weaknesses of innovation indicators, including R&D expenditure and patents and publications, see Kleinknecht et al. 2002). In contrast, website data appears to show R&D activity that is mid-process, downstream or customer-oriented, as firms have inherent marketing motives to report such activities on their websites. Again, noting that website data is self-reported, there is the possibility that firms might over-represent their activities in their websites (for example, claiming new product developments that are perhaps neither new nor innovative).

#### Currency

Website data is potentially more current than other data sources. Whilst some firms use websites as placeholders and update them infrequently, most firms tend to add information to their websites constantly and delete information that they think no longer represents their firm (although many older or deleted pages can be retrieved via the Wayback Machine). In contrast, financial data is often already outdated at the time of its release. For instance, firms in the UK are required to submit their accounts to Companies House within 6–9 months (depending on the legal status) of the accounting reference date. Moreover, it usually takes a further few months for this data to be reported in financial databases such as FAME. Similarly, publications, and particularly patents, often take a long time before they are publicly available.

#### Volatility (frequency)

The relatively higher currency of the website data is also related to the frequency of change (volatility). Website data, as a category, is refreshed every day, as opposed to reported financial data, which changes every 12 months. Patents and publication data and government support data change relatively more frequently than financial data but less than website data.

#### Consistency

Agreement between the components of the data is an important data quality dimension. There are issues with all of the data sources we discuss. International guidelines and principles are promulgated on what to include in R&D expenditures (see, for example, the Frascati guidelines on the measurement of science and technological activities in OECD 2002). However, accounting practices differ, particularly among smaller firms. This introduces a certain bias in making the comparison of R&D expenditure between different firms. Similarly, patents and publications vary greatly in terms of quality, which in turn make comparison problematic. R&D grants data is more consistent because of government procedures and regulations. Website data is the least consistent of all the sources considered as the motivations for posting information vary greatly between different firms. Companies may vary in what they choose to disclose and in the way that they report information on their web sites. At the same time, the public nature of websites allows false information to be exposed (and this would not be helpful for firms that seek to maintain their business reputations).

#### Interpretability

There is a wealth of literature explaining the meaning of enterprise financial data and government grant data. Patent and publication data are also structured and the components of this data are straightforward to understand. However, website data is often difficult to interpret and is sensitive to methodological choices. As we discussed earlier, changes in keywords and search methods used to capture R&D activity do affect the results. Care needs to be taken in methods used to make sense of website data, and it is generally useful to build in multiple iterative steps to review and refine the procedures used.

#### Accessibility

Enterprise financial and government support data is often more difficult to access than patent, publication or publicly available website data. In our case, UK government grant support data was recently made available but in many other countries this kind of data is not available to the public. Proprietary databases (such as FAME or the Web of Knowledge) require subscriptions (although universities often subscribe to such databases and make them available for affiliated researchers and students).

#### Handling

Website data is more difficult to handle as it is initially in an unstructured form, whilst the semi-structured nature of patent and publication data makes the analysis relatively easier. The methodology outlined in previous sections demonstrates that handling website data is at present a complex, multistep process. Financial and government support data is the easiest in terms of analysis due to established methods and procedures.

#### Amount

Websites include large amounts of information, in contrast to financial and government support data, which often include a limited number of well-defined variables. Patent and publication data is also sizeable compared to financial data but still relatively smaller than website data. As illustrated in Table 1, which shows the number of websites, webpages and noun phrases in our data set, in 2012 our dataset included over 50,000 webpages and 10 million noun phrases. The substantial quantity of website data provides opportunities in terms of flexibility (as noted below) in what can be analyzed, although sheer size can present problems of data management and operationalization.

#### Flexibility

Due to its unstructured nature, website data is adaptable to a range of purposes. Different keyword sets and variables can be extracted from websites depending on the research goals. Semi-structured patent and publication data is less flexible than the website data, whilst structured financial and government support data is the least flexible. In our dataset, both financial and publication and patent data is used to investigate the R&D activity. There are other categories of variables, including those related to manufacturing strategy, linkages, investment strategy and policy, which can be mined and analyzed.

## Conclusions

Website data is finding increasing applications in social science, including in innovation studies. Website data has potential advantages over other data sources on aspects such as coverage, currency, accessibility, quantity and flexibility, whilst there are potential issues about its consistency, interpretability and handling.

In this article, we discussed a particular case in which we operationalized website-based variables to understand R&D activity, amongst others. The processes of data retrieval, preparation, cleaning and analysis for web content data are more complex compared to conventional data sources. Care in the interpretation of the website data is particularly important. As shown in the paper, approaches to searching that are too simplistic and overlook context can lead to false positives or incorrectly dropping instances. Studies using website data should conduct extensive sensitivity analyses and also make their methodology transparent.

However, once these steps are performed, website data promises valuable complementary and new insights. We established that for R&D activity the correlation between website-based variables and non-website-based variables was not significant. This reflects the different facets of R&D activity shown on websites when compared with other conventional R&D data sources. Website data frequently highlights downstream or customer-focused aspects of R&D processes, particularly those related to technology and product development, as firms announce their new R&D projects and activities on their websites. In contrast, the annual R&D expenditure for firms, which is the financial data for R&D obtained from the FAME database, essentially gives information about one of the inputs for R&D. Furthermore, financial data is annually aggregated data, as opposed to website data, which tends to show discrete individual R&D activities. R&D expenditure is not the only input to the R&D process and subsequently to innovation (Smith 2005), thus a lack of correlation between R&D expenditure and the R&D process indicated by the website data might also be due to the relative importance of non-financial inputs to the R&D process of the firms in our sample. The same logic applies to the R&D grants provided by the government, as they are another indicator of R&D expenditure and thus an input indicator as well.

The website-based indicator of R&D is also not significantly correlated with patents and publications. Patents and publications show important formal results of R&D activities but not the other complementary processes that bring the results of formal R&D activities to the marketplace (and which are often not well-captured by conventional data sources). It is well documented that there are a number of factors that influence patenting and publishing behavior and firms may decide not to patent or publish the outputs of their R&D activity. Firms may also choose not to place all details of their R&D activities on public websites, but they can generally describe what they are doing for the benefit of customers (real and potential) without revealing extensive technical details to their competitors. This helps to explain the lack of correlation between the website-based indicator of R&D activity and patent and publication based R&D output. The lack of correlation between different conventional indicators of R&D, their relative strengths and weaknesses, and the particular biases associated with them is already well documented in the literature (see, for example, Kleinknecht et al. 2002). The repeated conclusion in many studies is to recommend use of indicators that are appropriate to the purpose, context and particular issues being investigated. Our conclusions augment the previous literature by explicating a framework of relative strengths and weaknesses of website data. This can help in making choices about which data sources and approaches to use in a particular study.

We conclude that website data is promising both as a complementary and an additional source of enterprise information that is useful in studying innovation. There are caveats and limitations to interpreting enterprise website information, and the handling of large website datasets presents its own set of issues. Using website data needs particular technical skills, including skills which are different from those used in the handling of conventional and well-established data sources. In the near future, improved software and more easily manageable searching and analytical methods may make it easier to handle large-scale data mining of web and other online sources. However, researchers in innovation studies (as in other fields) should continue to experiment with using website data: this exploration is likely to open up new ways to generate enhanced understandings of complex innovation processes.

## Footnotes

1 Calculated by the authors from data on UK companies, industry sectors, employment and website addresses contained in FAME (2014). Search conducted on June 30, 2014.
